# Genetic determinants of disease severity in the myotonic dystrophy type 1 OPTIMISTIC cohort

**DOI:** 10.1212/WNL.0000000000008056

**Published:** 2019-09-03

**Authors:** Sarah A. Cumming, Cecilia Jimenez-Moreno, Kees Okkersen, Stephan Wenninger, Ferroudja Daidj, Fiona Hogarth, Roberta Littleford, Gráinne Gorman, Guillaume Bassez, Benedikt Schoser, Hanns Lochmüller, Baziel G.M. van Engelen, Darren G. Monckton, on behalf of the OPTIMISTIC Consortium

**Affiliations:** From the Institute of Molecular, Cell and Systems Biology (S.A.C., D.G.M.), University of Glasgow; Institute of Genetic Medicine (C.J.-M., H.L.) and Institute of Neurosciences (G.G.), Newcastle University, Newcastle upon Tyne, UK; Department of Neurology (K.O., B.G.M.v.E.), Donders Institute for Brain Cognition and Behaviour, Radboud University Medical Centre, Nijmegen, the Netherlands; Department of Neurology (S.W., B.S.), Friedrich-Baur-Institute, Ludwig-Maximilians-Universität München, Munich, Germany; Neuromuscular Reference Centre (F.D., G.B.), Assistance Publique-Hôpitaux de Paris, France; and Tayside Clinical Trials Unit (F.H., R.L.), The University of Dundee, UK.

## Abstract

**Objective:**

To evaluate the role of genetic variation at the *DMPK* locus on symptomatic diversity in 250 adult, ambulant patients with myotonic dystrophy type 1 (DM1) recruited to the Observational Prolonged Trial in Myotonic Dystrophy Type 1 to Improve Quality of Life—Standards, a Target Identification Collaboration (OPTIMISTIC) clinical trial.

**Methods:**

We used small pool PCR to correct age at sampling biases and estimate the progenitor allele CTG repeat length and somatic mutational dynamics, and AciI digests and repeat primed PCR to test for the presence of variant repeats.

**Results:**

We confirmed disease severity is driven by progenitor allele length, is further modified by age, and, in some cases, sex, and that patients in whom the CTG repeat expands more rapidly in the soma develop symptoms earlier than predicted. We revealed a key role for variant repeats in reducing disease severity and quantified their role in delaying age at onset by approximately 13.2 years (95% confidence interval 5.7–20.7, 2-tailed *t* test *t* = −3.7, *p* = 0.0019).

**Conclusions:**

Careful characterization of the *DMPK* CTG repeat to define progenitor allele length and presence of variant repeats has increased utility in understanding clinical variability in a trial cohort and provides a genetic route for defining disease-specific outcome measures, and the basis of treatment response and stratification in DM1 trials.

Myotonic dystrophy type 1 (DM1) is a highly variable autosomal dominant inherited disorder affecting individuals of both sexes and all ages.^1^ DM1 is caused by the expansion of a CTG repeat in the *DMPK* gene that varies in length from under 40 repeats in the general population to more than 1,000 repeats in some patients.^2–7^ Longer CTG tracts are associated with more severe disease and earlier age at onset.^8–11^ However, genotype to phenotype relationships determined using the traditional diagnostic test have low predictive value and the International Myotonic Dystrophy Consortium recommends that families are not offered predictive phenotypic information based on the size of the CTG repeat.^12^

Expanded disease-causing alleles are highly unstable and usually increase in length during intergenerational transmission,^8,13–15^ explaining the decrease in age at onset of 20–30 years per generation typically observed in DM1 families.^16^ The expanded CTG is also highly unstable in the soma in a process that is expansion-biased, age-dependent, and tissue-specific.^17–24^ Thus, the average length of the CTG repeat measured using the traditional diagnostic depends on the age at which the sample was taken. Previously, we demonstrated that a sensitive small pool PCR (SP-PCR) analysis of blood DNA could be used to estimate the progenitor allele length (ePAL), i.e., the length of the CTG repeat transmitted to the individual by the affected parent.^18^ In addition, we demonstrated that ePAL could reduce the confounding effects of age at sampling inherent in the traditional test and we could dramatically improve the ability to predict age at onset in DM1.^25,26^

Observational Prolonged Trial in Myotonic Dystrophy Type 1 to Improve Quality of Life—Standards, a Target Identification Collaboration (OPTIMISTIC) was a large international, multicenter, model-based randomized trial designed to compare the utility of tailored cognitive behavioral therapy (CBT) with an optional graded exercise plan against standard care.^27,28^ In order to provide additional insight into the genetic basis of variability in DM1 and as a route toward genetic stratification in clinical trials, we sought to estimate the progenitor allele length and define the repeat dynamics of the DM1 CTG repeat in the blood DNA of participants recruited to OPTIMISTIC.

## Methods

### Standard protocol approvals, registrations, and patient consents

Full details of the OPTIMISTIC protocol, including power calculations for sample size, have been reported previously (Clinicaltrials.gov, NCT02118779).^27^ All participants were recruited with informed consent. The protocol was approved by the National Research Ethics Service Committee North East—Sunderland (UK), the Comite de Protection des Personnes ile de France V (France), the Ethikkommission bei der LMU München (Germany), and the Concernstaf Kwaliteit en Veiligheid Commissie Mensgebonden Onderzoek Regio Arnhem-Nijmegen (Netherlands).

### Phenotypic data

Ambulant adult patients (>18 years of age) with genetically proven DM1 capable of providing informed consent with severe fatigue (Checklist Individual Strength–Fatigue [CIS-Fatigue] score ≥35) were recruited between April 2014 and May 2015 through 4 clinical centers: Nijmegen, the Netherlands; Paris, France; Munich, Germany; and Newcastle, United Kingdom (n = 255 [46.3% female] [118/255]).^27,28^ Exclusion criteria included neurologic or orthopedic comorbidity possibly influencing the intervention or outcomes; use of psychotropic drugs (except modafinil, Ritalin, and antidepressants, where the dosing regimen was stable for at least 12 months prior to screening); severe depression; participation in another interventional clinical trial; or inability to complete study questionnaires. Age at onset of DM1 symptoms was obtained during the trial recruitment screening visit in answer to the question “At what age did the first medical problems occur that may be related to your myotonic dystrophy?” With the exception of patients recruited through Munich, age at sampling was calculated based on known date of birth and the date of visit 2, at which the blood sample was provided. As a local regulatory requirement, date of birth was not recorded for participants recruited in Munich, therefore the age at sampling used was self-reported by participants at visit 2. Additional baseline (visit 2) clinical measures were obtained as described in the OPTIMISTIC protocol and reported previously.^27,28^ The majority of participants (n = 225) were not known to be related. Eight parent–offspring pairs, 4 sibling pairs, and 3 trios of 2 siblings and 1 offspring were also recruited.

### DNA samples

A 10-mL venous blood sample was taken from OPTIMISTIC participants at baseline (time point V2). Blood was collected into EDTA tubes and stored locally at −80°C. Frozen blood was shipped on dry ice to the Newcastle Biobank for Research of Neuromuscular Disorders,^29^ where DNA was isolated and shipped to the University of Glasgow. Baseline DNA samples were available from 250 out of 255 OPTIMISTIC participants. Historical DNA samples from OPTIMISTIC participants collected during routine molecular diagnosis (from 6 months to 21 years previously) were available for 71 participants and obtained from local diagnostic laboratories via the Newcastle Biobank. Historical DNA samples were not available for the 5 participants without baseline DNA samples.

### SP-PCR and AciI digestion

SP-PCR was carried out using flanking primers DM-C and DM-DR as previously described^18,30^ using Custom PCR Master Mix (Thermo Fisher Scientific [Waltham, MA] #SM-0005) supplemented with 69 mM 2-mercaptoethanol. Taq polymerase (Sigma-Aldrich UK, Gillingham) was used at 1 unit per 10 µL. Where required, reactions were supplemented with 10% DMSO and the annealing temperature reduced to 63.5°C. PCR products were digested with AciI (New England Biolabs UK, Hitchin) in accordance with the manufacturer's instructions. DMSO was removed prior to AciI digestion using the QIAquick (Qiagen, Venlo, the Netherlands) PCR purification kit. DNA fragments were resolved by agarose gel electrophoresis and Southern blot hybridized as described.^18,30^ Autoradiographic images were scanned and ePAL and modal allele lengths were estimated from the lower boundary^18^ and the densest part of the expanded allele distribution respectively by comparison against the molecular weight ladder, using CLIQS 1D gel analysis software (TotalLab UK, Newcastle upon Tyne).

### Repeat-primed PCR (RP-PCR)

RP-PCR was performed at the 5′-end of the repeat tract using 1 μM DM-A, 0.1 μM TAG-CAG_5_, and 1 μM TAG primer and at the 3′-end of the repeat tract using 1 μM DM-DR, 0.1 μM TAG-CTG_5_, and 1 μM TAG primer, as described previously.^31^

### Statistical analysis

Statistical analyses were undertaken in R (version 3.4.3)^32^ using RStudio (version 1.0.153).^33^ There were no repeated measures and all individuals are represented once in each statistical analysis. All statistical analyses were 2-sided. Log transformations of the age at onset, degree of somatic instability, and Stroop interference score were used to better approximate a normal distribution and minimize the influence of extreme values. Participants with missing data were excluded from relevant analyses. Multiple linear regressions were performed using the lm function from the R Stats package. In all regression analyses, we report the adjusted *r*^2^, the *p* value for the overall model, and the coefficient (β) and *p* value for each measure. Model selection was based on the Akaike information criterion for each model using a backwards stepwise selection procedure implemented using the step function in R.^34^ Confidence intervals (CI) (95%) for point estimates were calculated where appropriate.

### Data availability

The de-identified participant genetic data presented here are available online (datadryad.org/review?doi=doi:10.5061/dryad.t063q70). Requests to access the de-identified participant data from the main OPTMISTIC trial should be addressed to the trial chief investigator Dr. Baziel van Engelen (baziel.vanengelen@radboudumc.nl).

## Results

### Estimation of progenitor and modal allele CTG repeat lengths in the OPTIMISTIC cohort

**Figure 1:**
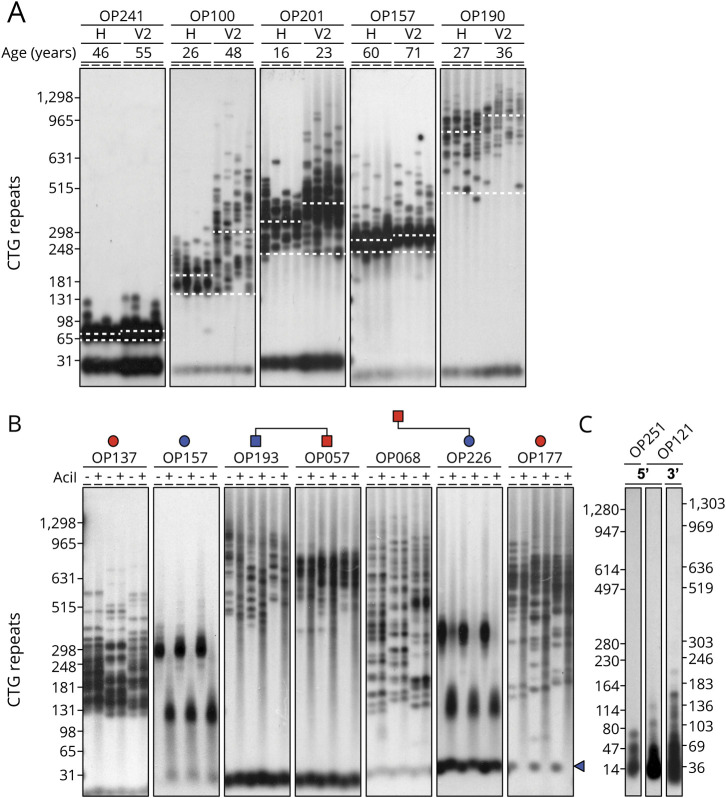
Estimation of progenitor and modal CTG repeat length and detection of AciI-sensitive variant repeats (A) Shown are representative small pool PCR (SP-PCR) analyses of repeat length variation at the *DMPK* CTG repeat in blood DNA from 5 individuals. For each participant, the analysis of 2 DNA samples is shown: historical sample taken at time of initial diagnosis (H) and at baseline (V2). The age at sampling for each sample is indicated in years. The approximate positions of the molecular weight standards converted into the number of CTG triplet repeats are shown on the left. The position of the estimated progenitor allele length for each individual is indicated with the lower dashed white line. The modal allele length at each time point is indicated by the upper dashed white line. For each sample, 4 replicate PCRs were performed with ∼180–300 pg DNA. (B) Shown are representative SP-PCR and AciI digestions of the *DMPK* CTG repeat in blood DNA from 7 individuals. For each participant, 3 replicate PCRs were performed with ∼500 pg DNA, digested (+) or undigested (−) with AciI, resolved by agarose gel electrophoresis and Southern blot hybridized with a repeat unit probe. The approximate positions of the molecular weight standard converted into the number of CTG triplet repeats of undigested products is shown on the left. For each participant the presence (blue) or absence (red) of AciI-sensitive variant repeats within the expanded allele is indicated by filled pedigree symbols. Note OP177 contains AciI-sensitive variant repeats within the non-disease-associated allele as indicated by the blue triangle. (C) Detection of expanded alleles using repeat-primed PCR (RP-PCR). Shown are 5′- and 3′-RP-PCR assays for 2 participants (OP251 and OP121) in which we were not able to amplify the expanded allele using flanking primers. For both participants, a ladder of products extending beyond 50 repeats was observed using RP-PCR, confirming the presence of a disease-causing expansion. Notably, the ladders were discontinuous, consistent with the presence of variant repeat blocking amplification from the (CAG)_5_ or (CTG)_5_ repeat primer at some positions within the array. The approximate positions of the molecular weight standard converted into the number of triplet repeats are shown.

In order to investigate genetic variation in the OPTIMISTIC cohort, we used SP-PCR^18^ to determine the ePAL, modal allele length at recruitment, and where available (71 participants), modal allele length at molecular diagnosis for the expanded CTG repeat *DMPK* allele (figure 1A). An expanded *DMPK* CTG repeat allele was readily detected in 241 out of 250 participants using our standard SP-PCR procedure. In an additional 7 participants, an expanded allele was only detected after the addition of 10% DMSO to the PCR, consistent with the presence of GC-rich variant repeats.^31^ In one of these individuals, even in the presence of DMSO, only a small number of expanded alleles was successfully amplified relative to the non-disease-associated allele and it was not possible to define the ePAL or modal allele length. In 2 remaining individuals, we could not successfully amplify an expanded allele, although in both cases it was possible to amplify a single nonexpanded allele, consistent with the presence of a nonamplifiable expanded allele containing a large number of GC-rich variant repeats (see below for additional analyses). In the cases where we were able to obtain a historical DNA sample, the modal allele length had nearly always noticeably increased in size between the time of diagnosis and recruitment to the trial (figure 1A). As expected, it was easier to estimate ePAL from the diagnostic DNA sample obtained at an earlier age, confirming the value of these historical DNA samples.

### Identification of variant repeats within the CTG arrays of the OPTIMISTIC cohort

Although the majority of patients with DM1 likely inherit a pure CTG repeat expansion at the *DMPK* locus, it is known that a subset of patients contains variant repeat interruptions within the disease allele.^31,35^ Such variant repeats, most commonly CCG and CGG, have been shown to reduce both germline and somatic instability^31^ and appear to be associated with less severe symptoms.^31,35–37^ Thus, we used SP-PCR to amplify the CTG repeat tract and test for the presence of variant repeats by post-PCR digestion with AciI (figure 1B). AciI recognizes the sequence CCGC and cleaves both CCG and CGG variant repeats.^31^ Using this approach, we identified variant repeats in 19 out of 248 expanded alleles (7.7%). This included all the samples that required the addition of DMSO to facilitate PCR amplification. For the 2 samples for which we could not amplify the expanded allele, we used RP-PCR^31,38,39^ to confirm the presence of an expanded allele (figure 1C). The presence of gaps within the repeat ladder of these 2 participants is consistent with the existence of variant repeats within the expanded allele. Thus, overall, the total frequency of participants identified with variant repeats in the expanded allele was 8.4% (21/250 [13/21 female], 95% CI 5.4%–12.7%). This included 1 pair of siblings, in both of whom we detected variant repeats. Interestingly, we also detected variant repeats in the daughter of a male transmitting parent in whom we did not detect variant repeats, consistent with de novo gain of variant repeats (figure 1B). Similarly, we also detected variant repeats in only 1 of a pair of siblings, consistent with either a de novo gain or loss of variant repeats (figure 1B). Although the proportion of participants with variant repeats appeared to differ between centers (Munich 5/65 [7.7%, 95% CI 2.8%–17.8%], Newcastle 4/52 [7.7%, 95% CI 2.5%–19.4%], Nijmegen 9/66 [13.6%, 95% CI 6.8%–24.8%], Paris 3/67 [4.5%, 95% CI 1.2%–13.4%]), these differences were not statistically significant (Fisher exact test, *p* = 0.32). In one participant, we detected variant repeats in a 34-triplet large non-disease-associated allele (figure 1B).

### Age at onset correlations in the OPTIMISTIC cohort

**Figure 2:**
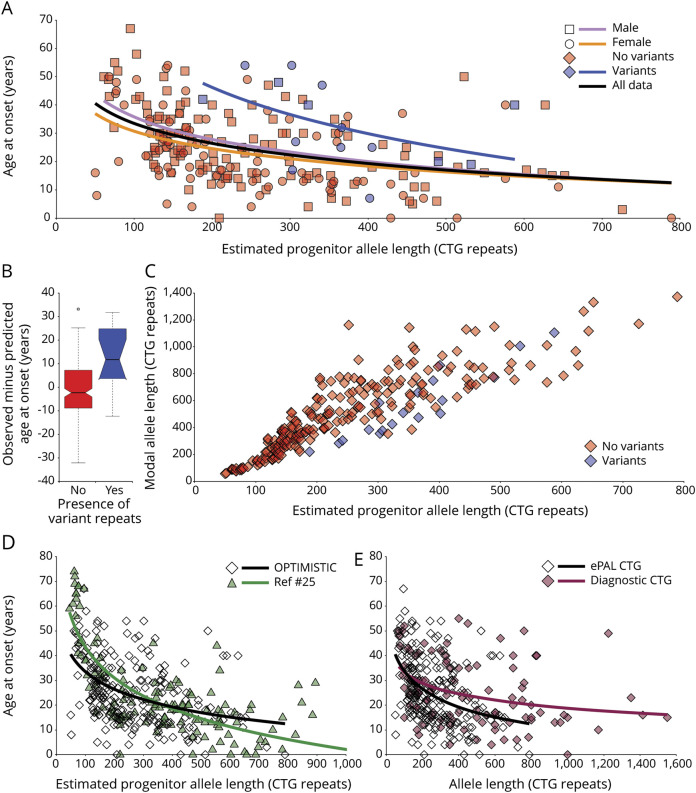
Age at onset and somatic instability correlations (A) Age at onset is highly correlated with estimated progenitor allele length (ePAL). The scatterplots show the relationship between ePAL and age at onset. The relevant line of best fit under a logarithmic model for female individuals (circles, orange line), male individuals (squares, lavender line), and sex-averaged for all individuals (black line, model 1a) (table 1) are shown. Individuals with (blue) and without (red) AciI-sensitive variant repeats are also depicted, along with the logarithmic regression line for AciI-sensitive variant repeat carriers (blue line). (B) Effect of variant repeats on age at onset. Shown are boxplots for the difference in observed age at onset minus predicted age at onset for Observational Prolonged Trial in Myotonic Dystrophy Type 1 to Improve Quality of Life—Standards, a Target Identification Collaboration (OPTIMISTIC) participants with (yes, blue) and without (no, red) AciI-sensitive variant repeats in their expanded *DMPK* allele. Predicted age at onset was derived using model 1a (*Age*_*o*_ = 80.3 + [−23.4 × log(ePAL)]) (table 1). The mean difference in age at onset for participants carrying AciI-sensitive variant repeats was 13.2 years (95% confidence interval [CI] 5.7 to 20.7, 2-tailed *t* test *t* = −3.7, *p* = 0.0019). The bottom and top of the box are the lower and upper quartiles, respectively. The band near the middle of the box is the median and the notches approximate to the 95% CI for the medians. The whiskers represent the full range of observations bounded by an upper limit equal to the upper quartile plus 1.5× the interquartile range, and a lower limit equal to the lower quartile minus 1.5× the interquartile range. Any points outside these bounds are displayed individually as small circles. (C) Modal allele length, ePAL, and presence of AciI variant repeats. The scatterplots show the relationship between ePAL and modal allele length at the V2 time point. Individuals with (blue) and without (red) AciI-sensitive variant repeats are indicated. (D) Sampling bias in the OPTIMISTIC cohort. The scatterplots show the relationship between ePAL and age at onset for the OPTIMISTIC cohort (open diamonds, black line) and a family-based DM1 population characterized by Morales et al.^25^ (green triangles and line). The relevant line of best fit under a logarithmic model (model 1a, table 1) is shown for each population (for the OPTIMISTIC cohort n = 222, *r*^2^ = 0.178, *p* = 3.1 × 10^−11^ and for Morales et al., n = 137, *r*^2^ = 0.640, *p* < 2.2 × 10^−16^). Note that the Morales et al. cohort has more very mildly affected participants with small expansions, and more severely affected patients with large expansions. (E) ePAL is more informative than the diagnostic measure in predicting age at onset. The scatterplots show the relationship between age at onset and ePAL (ePAL, open diamonds and black line) and the diagnostic allele length (diagnostic CTG, maroon diamonds and line) for the OPTIMISTIC cohort. The relevant line of best fit under a logarithmic model (model 1a, table 1) is shown for each CTG measure (for ePAL n = 222, *r*^2^ = 0.178, *p* = 3.1 × 10^−11^ and for diagnostic CTG n = 105, *r*^2^ = 0.123, *p* = 0.00014).

Self-reported age at onset of symptoms was available for 229 participants and we used linear regression modeling to investigate genotype–phenotype relationships. As expected,^25,26,40^ ePAL was a major modifier of age at onset (figure 2A and table 1, model 1a, *r*^2^ = 0.178, *p* = 3.1 × 10^−11^). The presence of variant repeats was also highly significantly (*p* = 2.2 × 10^−5^) associated with a later age at onset (figure 2A and table 1, model 1b, *r*^2^ = 0.240, *p* = 3.4 × 10^−14^). In order to better quantify these effects, we calculated the age at onset predicted by ePAL alone (i.e., model 1a) and compared this to the observed age at onset. The mean difference in age at onset between individuals with and without variant repeats was 13.2 years (95% CI 5.7 to 20.7, 2-tailed *t* test *t* = −3.7, *p* = 0.0019, figure 2B). Recently, it has been reported that there are substantial sex-dependent differences in the relative incidence and severity of some aspects of DM1.^41^ Similarly, we recently reported some evidence for sex-dependent genotype–phenotype correlations in age at onset, although these appeared to be driven largely by an excess of mildly affected transmitting grandfathers.^40^ Here, sex was revealed as having a marginally significant effect (*p* = 0.015) on age at onset (table 1, model 1c, *r*^2^ = 0.257, *p* = 1.2 × 10^−14^).

**Table 1:**
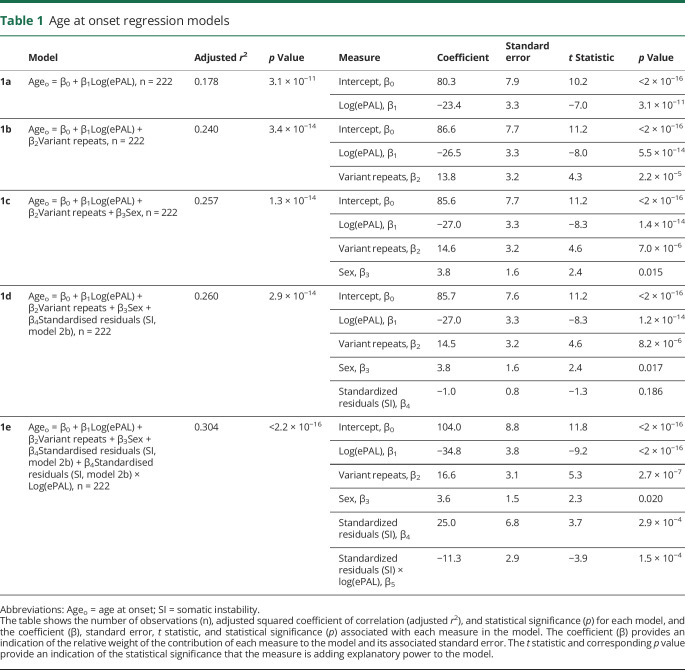
Age at onset regression models

### Determinants of somatic instability in the OPTIMISTIC cohort

We defined the degree of somatic instability as the difference between ePAL and the modal allele at recruitment (time point V2). As expected,^25,40^ the major determinants of the degree of somatic instability were ePAL (figure 2C), age at sampling, and an interaction between them (table 2, model 2a, *r*^2^ = 0.560, *p* < 2.2 × 10^−16^). Previously, we also demonstrated in one family that the presence of variant repeats reduced the degree of somatic instability.^31^ Likewise, here the presence or absence of variant repeats (figure 2C) was revealed as a highly significant (*p* = 4.9 × 10^−8^) additional measure in the regression model (table 2, model 2b, *r*^2^ = 0.610, *p* < 2.2 × 10^−16^). In contrast, sex of the participant (*p* = 0.48) was found not to improve the regression model (table 2, model 2c, *r*^2^ = 0.609, *p* < 2.2 × 10^−16^).

**Table 2:**
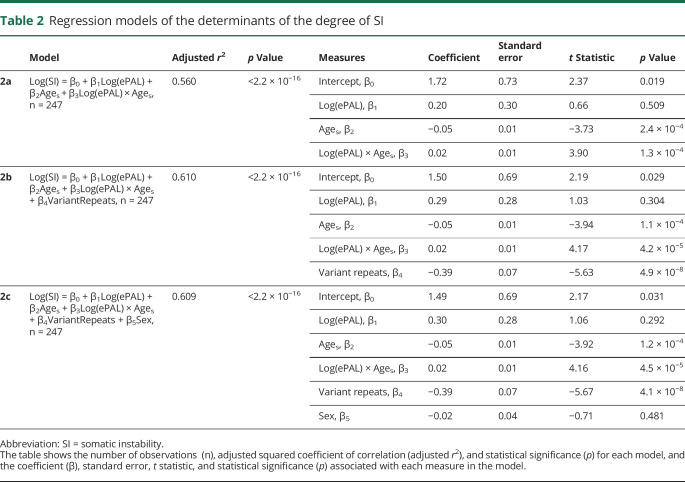
Regression models of the determinants of the degree of SI

### The role of somatic instability on age at onset in the OPTIMISTIC cohort

As longer inherited allele lengths precipitate an earlier age at onset in DM1, and as somatic instability is highly biased toward expansions, it seems logical to expect that individual-specific differences in the rate of expansion should modify age at onset. Indeed, we previously established evidence for such an effect in a mixed cohort of patients with DM1 from Scotland, the United States, and Costa Rica,^25^ and confirmed this effect in a large Costa Rican cohort.^40^ In the OPTIMISTIC cohort, incorporating the standardized residuals of somatic instability derived from model 2b into the age at onset model (table 1, model 1d, *r*^2^ = 0.260, *p* = 3.0 × 10^−14^) improved the *r*^2^ value slightly from model 1c, but the standardized residuals of somatic instability did not reach statistical significance as a measure (*p* = 0.19). However, when the standardized residuals of somatic instability were included with an interaction with ePAL, the model was improved further (table 1, model 1e, *r*^2^ = 0.304, *p* < 2.2 × 10^−16^) and both the standardized residuals of somatic instability alone (*p* = 2.9 × 10^−4^) and the interaction with ePAL (*p* = 1.5 × 10^−4^) were revealed as highly significant measures.

### Genetic correlations with progressive phenotypes in the OPTIMISTIC cohort

**Figure 3:**
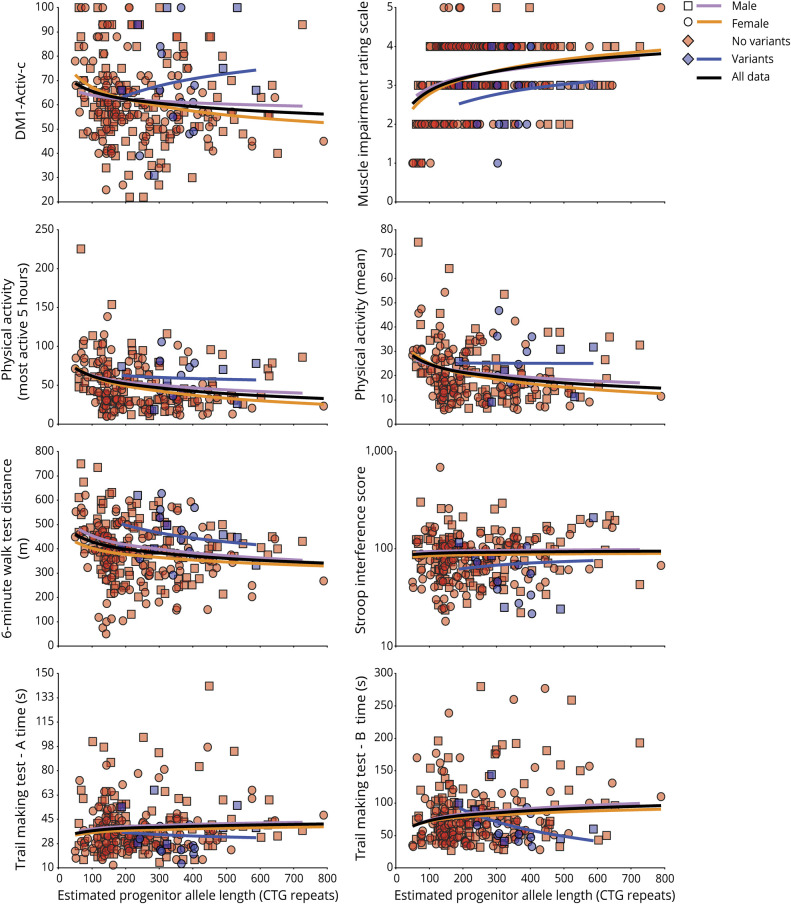
Estimated progenitor allele length (ePAL) and variant repeat correlations with age-dependent phenotypes The scatterplots show the relationship between ePAL and a number of age-dependent phenotypes. The relevant line of best fit under a logarithmic model (dependent variable = β_*0*_ + β_1_Log[ePAL]) for female individuals (circles, orange line), male individuals (squares, lavender line), and sex-averaged for all individuals (black line) are shown. Individuals with (blue) and without (red) AciI-sensitive variant repeats are also depicted, along with the logarithmic regression line for AciI-sensitive variant repeat carriers (blue line).

As part of the OPTIMISTIC protocol, several DM1 phenotypes were also quantified at baseline using a variety of direct and indirect (i.e., questionnaires) assessment tools.^27^ To investigate the genotype–phenotype relationships of these progressive phenotypes, we applied a model selection process incorporating ePAL, residual variation in somatic instability, variant repeats, sex and age at baseline, and interactions between ePAL, residual variation in somatic instability, and age at baseline (table 3). Highly significant associations with effect sizes comparable to those observed with age at onset were revealed for many measures (table 3 and figure 3), including the Muscle Impairment Rating Scale (MIRS*, r*^2^ = 0.32, *p* = 2.6 × 10^−19^), the OPTIMISTIC primary outcome, the DM1-Activ-c score (*r*^2^ = 0.23, *p* = 2.9 × 10^−13^), and key secondary outcomes such as the 6-Minute Walk Test (6-MWT, *r*^2^ = 0.31, *p* = 2.3 × 10^−18^), physical activity (accelerometry, mean, *r*^2^ = 0.25, *p* = 3.9 × 10^−10^, and most active 5-hour, *r*^2^ = 0.25, *p* = 8.9 × 10^−12^), the Trail-Making Test (TMT-A, *r*^2^ = 0.23, *p* = 2.2 × 10^−14^, and TMT-B, *r*^2^ = 0.24, *p* = 4.5 × 10^−12^), and the Stroop interference score (*r*^2^ = 0.21, *p* = 2.8 × 10^−12^). Moderate associations were observed for the Adult Social Behavior Questionnaire (ASBQ, *r*^2^ = 0.11, *p* = 1.6 × 10^−4^), the Apathy Evaluation Scale–Clinician Version (*r*^2^ = 0.10, *p* = 5.1 × 10^−5^), the Fatigue and Daytime Sleepiness Scale (FDSS) (*r*^2^ = 0.09, *p* = 2.9 × 10^−5^), the Myotonic Dystrophy Health Index total score (*r*^2^ = 0.09, *p* = 3.4 × 10^−5^), and Self-Efficacy–Scale 28 for Fatigue (*r*^2^ = 0.07, *p* = 2.2 × 10^−4^). Only weak or statistically nonsignificant associations were observed for a number of measures, including for van Sonderen Social Support List (SSL) (SSL—Discrepancies [SSL-D], SSL—Interactions [SSL-I], and SSL—Negative Interactions [SSL-N]), CIS-Fatigue, the McGill Pain Questionnaire, the Sickness Impact Profile, and the Beck Depression Inventory—fast screen (BDI-fs), all *r*^2^ < 0.05, *p* > 0.01, and n > 150 (table 3). For all of the highly associated measures (*r*^2^ > 0.15), age, ePAL, and presence of variant repeats were revealed as important explanatory variables (table 3). The relative degree of somatic mosaicism was also an important explanatory variable for all bar the Stroop interference score and TMT-A. Sex was also an important modifier for the 6-MWT and physical activity (accelerometry, mean, and most active 5-hour) (table 3).

**Table 3:**
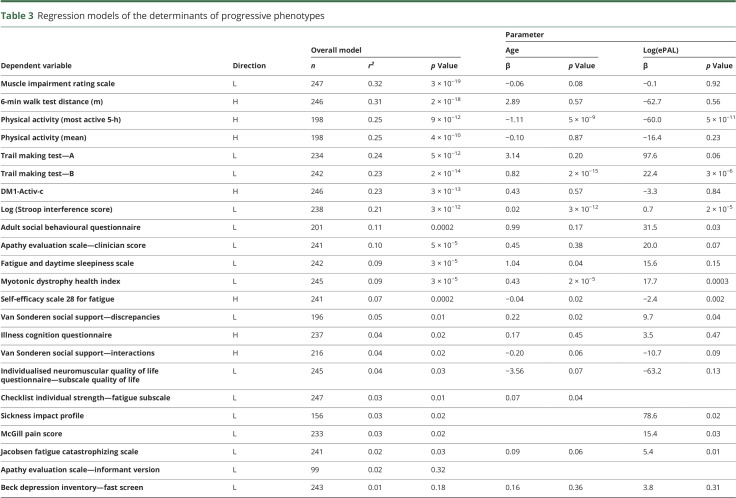

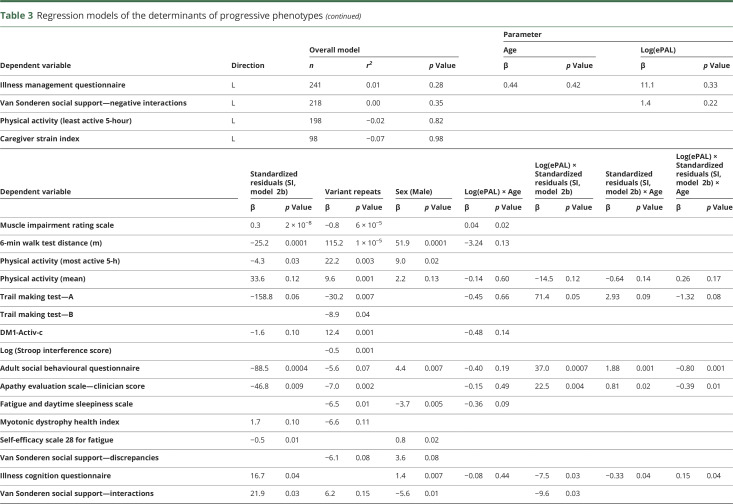

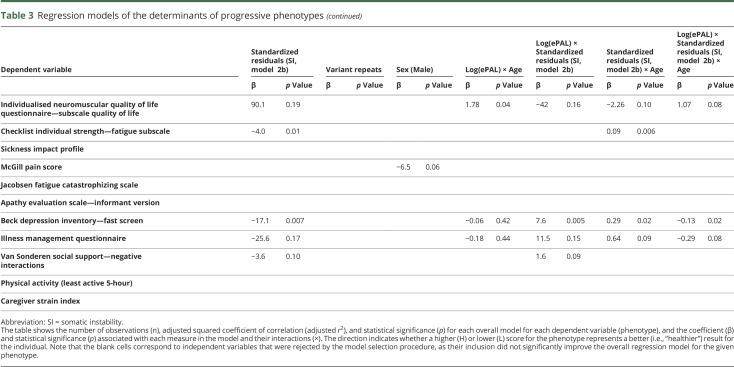
Regression models of the determinants of progressive phenotypes

## Discussion

We analyzed the length and dynamics of the disease-causing CTG repeat expansion in the *DMPK* gene to reveal the primary genetic determinants of symptomatic variation in the OPTIMISTIC cohort. As expected, we were able to establish ePAL as a major determinant of self-reported age at onset (table 1, model 1a). However, it is notable that the proportion of variance in age at onset explained by ePAL (*r*^2^ = 0.178) was much lower than observed in previous studies (e.g., *r*^2^ = 0.640 in Morales et al.^25^). This is attributable to the eligibility constraints for recruitment to OPTIMISTIC that dictated that participants must be aged at least 18 years, have severe fatigue (CIS-Fatigue severity score ≥35), but nonetheless retain the ability to walk independently, and be able to provide informed consent and be motivated to undergo CBT. Thus, OPTIMISTIC has fewer very mildly affected participants with small expansions, and fewer severely affected patients with very large DM1 expansions (figure 2D). Selection of moderately affected patients is likely to be a common feature of many of the early DM1 trials, suggesting that the genetic characteristics and phenotypic relationships of the OPTIMISTIC cohort will likely be representative of other DM1 trial cohorts. Although age at onset is an important aspect of the disease phenotype, it is somewhat subjective in its assessment as it may depend on patient recall, patient knowledge and relative cognition, and insight.^42^ Moreover, age at onset is unlikely in the near term to be an outcome measure in clinical trials. We have also revealed that ePAL is an important contributor to multiple age-dependent phenotypes that were also assessed as part of the OPTIMISTIC protocol, including key measures of muscle function and activity such as the MIRS, 6MWT, DM1-Activ-c, and accelerometry data, and key CNS phenotypes such as the TMT and Stroop test. In contrast, other phenotypes such as SSL-D/I/N, CIS-Fatigue, McGill pain, and BDI-fs were not well-explained by genetic factors. It seems reasonable to assume that in general symptoms that are strongly associated with the causative mutation in DM1 are most directly linked to the underlying disease process and may be the most responsive to therapeutic interventions targeting the underlying pathology. It is also possible that some of the more subjective self-reported measures may also be compromised by reduced patient insight,^42^ further confounding genotype to phenotype associations.

For many years it was assumed that expanded alleles at the *DMPK* locus comprised pure CTG repeat arrays. However, it has become apparent that a subset of DM1 expansions contains variant repeats.^31,35,37,43,44^ Previous estimates for the frequency of such variants in unselected DM1 cohorts vary from ∼3% to 5%.^31,35–37^ Here, we determined the overall frequency of variant repeats as 8.4% of the total cohort (21/250). In the Nijmegen subcohort, the frequency of variants was 13.6% (9/66, 95% CI 6.8%–24.8%), while in the Paris subcohort it was only 4.5% (3/67, 95% CI 1.2%–13.4%). Although there was 1 pair of siblings from Nijmegen who shared variant repeats, none of the other participants from Nijmegen with variant repeat containing expanded alleles were known to be related to each other. Thus, chance sampling of one or a few large families segregating variant repeat alleles does not explain the higher frequency of variant repeat alleles observed in the Nijmegen subcohort. While it is possible that this may reflect some sort of site-specific recruitment bias, this difference was not statistically significant and may simply represent random sampling error. Regardless, the presence of variant repeats clearly influences disease severity, with individuals carrying such alleles having an age at onset delayed by an average of ∼13 years relative to that predicted using the 1a regression model. It is possible that the reduced disease severity observed in DM1 carriers of repeat alleles led to their overselection in OPTIMISTIC, conceivably mediated by a reduced neuropsychological effect of the disease and higher motivation among this subcohort. In addition to modifying age at onset, these data also reveal the protective effect of variant repeats on many of the progressive phenotypes likely to be outcomes in clinical trials. If variant repeat carriers are over-recruited, this may reduce sensitivity to correctly evaluate efficacy of a therapeutic intervention in a clinical trial. These considerations suggest that testing for the presence of variant repeats should be included in DM1 trial design.

Recently, it has been demonstrated that the absolute frequency and severity of different aspects of the complex DM1 phenotype are differentially expressed in male and female patients.^41^ Similarly, we have presented preliminary evidence that suggest there may also be subtle repeat-length-independent sex effects on age at onset.^40^ Here, we also observed a marginally significant sex effect (*p* = 0.015) when including sex as a measure in the age at onset model (table 1, model 1c). However, the direction of this effect was the opposite to that previously observed. These observations suggest sex has only a subtle effect on overall age at onset. Nonetheless, sex was revealed as an important cofactor for some phenotypes such as the 6MWT, the ASBQ, and the FDSS (table 3) and should be considered as an important factor in clinical trials.

We recently demonstrated that residual variation in somatic instability not accounted for by age at sampling and ePAL was inversely correlated with residual variation in age at onset not accounted for by ePAL, i.e., patients in whom the repeat expands more rapidly in the soma have earlier ages at onset than expected.^25,40^ Here, we have used SP-PCR to calculate the modal length change during the lifetime of the patient as a measure of somatic instability and shown variation in this measure is explained by the expected measures, i.e., ePAL, age at sampling, and a strong interaction between them (table 2, model 2a). In addition, we have confirmed that somatic instability is reduced by the presence of variant repeats. We have also provided additional evidence that somatic mutational dynamics directly modify disease severity, with residual variation in somatic instability accounting for approximately 4.7% of the total variation in age at onset and detectably contributing to many age-dependent phenotypes and confirming somatic expansion as an important therapeutic target.

Traditionally, DM1 is diagnosed using Southern blot hybridization of restriction digested genomic DNA. The low predictive value of this measure and additional complications mediated by a failure to fully consider the effects of age-dependent somatic expansion on interpreting intergenerational length changes prompted the International Myotonic Dystrophy Consortium to recommend that families not be offered predictive phenotypic information based on the number of CTG repeats.^12^ While this recommendation is not universally observed, this suggestion, coupled with the technical challenges of Southern blot hybridizations and the availability of a simple yes/no RP-PCR test,^39^ has led to a situation where many diagnostic laboratories do not even attempt to measure the number of CTG repeats. For example, within the OPTIMISTIC cohort, a diagnostic CTG repeat length was available for only 121 out of 255 participants. As expected, the predictive value of this measure was relatively low, the diagnostic CTG length measure accounting for only 12% of the variation in age at onset (figure 2E, n = 103, *r*^2^ = 0.123, *p* = 0.00014). This contrasts sharply with the ∼30% of variation accounted for by estimating progenitor allele length, quantifying somatic mosaicism, and determining the presence or absence of variant repeats (table 1, model 1e). These data suggest that it may be time to revisit the recommended diagnostic criteria for DM1 and the potential value of reporting more informative prognostic information to families by estimating progenitor allele length and testing for the presence of variant repeats.

We have defined the genetic characteristics of the *DMPK* expansion in the cohort of patients with DM1 recruited to the OPTIMISTIC clinical trial. These baseline data have already yielded important insights into genotype to phenotype relationships in DM1 and should provide a route to determining the possible effect of genotype on intervention response and a basis for genetic stratification of DM1 trial participants.

## Glossary

6-MWT: 6-Minute Walk Test
ASBQ: Adult Social Behavior Questionnaire
BDI-fs: Beck Depression Inventory—fast screen
CBT: cognitive behavioral therapy
CI: confidence interval
CIS-fatigue: Checklist Individual Strength Fatigue score
DM1: myotonic dystrophy type 1
ePAL: estimated progenitor allele length
FDSS: Fatigue and Daytime Sleepiness Scale
MIRS: Muscle Impairment Rating Scale
OPTIMISTIC: Observational Prolonged Trial in Myotonic Dystrophy Type 1 to Improve Quality of Life—Standards, a Target Identification Collaboration
RP-PCR: repeat-primed PCR
SP-PCR: small pool PCR
SSL: van Sonderen Social Support List
SSL-D: van Sonderen Social Support List—Discrepancies
SSL-I: van Sonderen Social Support List—Interactions
SSL-N: van Sonderen Social Support List—Negative Interactions
TMT: Trail-Making Test
